# Diversity and plant growth promotion potential of endophytic fungi isolated from hairy vetch in Japan

**DOI:** 10.3389/fpls.2024.1476200

**Published:** 2024-12-19

**Authors:** Parisa Taheri, Khondoker M. G. Dastogeer, Safirah Tasa Nerves Ratu, Dominic V. A. Agyekum, Michiko Yasuda, Yoshiharu Fujii, Shin Okazaki

**Affiliations:** ^1^ United Graduate School of Agricultural Science, Tokyo University of Agriculture and Technology, Fuchu, Japan; ^2^ Department of Plant Pathology, Bangladesh Agricultural University, Mymensingh, Bangladesh; ^3^ Institute of Global Innovation Research, Tokyo University of Agriculture and Technology, Fuchu, Japan; ^4^ Faculty of Agriculture, Tokyo University of Agriculture and Technology, Fuchu, Japan

**Keywords:** hairy vetch, endophytic fungi, diversity, symbiosis, plant growth promotion

## Abstract

Hairy vetch (*Vicia villosa* Roth), a leguminous plant with nitrogen-fixing ability, is used as a cover crop and has the potential to suppress weeds and plant diseases. The microbial composition, particularly fungal endophytes, which may be related to the beneficial functions of this crop, has not been previously studied. In this study, we analyzed the diversity and function of culturable fungal endophytes associated with hairy vetch from eight locations across Japan. Using the fungal culture method, a total of 2,186 isolates were recovered and assigned to 80 distinct internal transcribed spacer (ITS) genotypes, spanning 28 genera. The results demonstrated that geographic location and soil physicochemical properties significantly influence the community composition of the hairy vetch fungal endophytes. Soil pH showed a significant positive correlation with the abundance of *Penicillium*, which was the most dominant genus in all the sampling locations and tissues. The majority of the isolates promoted plant growth and colonized hairy vetch and soybean roots, significantly promoting the growth of hairy vetch and/or soybean plants. Among the isolates, *Penicillium griseofulvum* AKL25 and *Trichoderma koningii* AKR15 significantly enhanced both hairy vetch and soybean growth, respectively. Meanwhile, *Alternaria alternata* OGL80 significantly increased soybean growth, but it did not affect hairy vetch growth, indicating host specificity of the fungal endophytes. In conclusion, this study showed that soil properties and geographic location play a critical role in shaping the community composition and structure of endophytic fungi associated with hairy vetch. Additionally, the isolated fungi promoted hairy vetch and soybean growth, with a host preference. Furthermore, this study revealed that a novel endophytic fungus, *P. griseofulvum* AKL25, which has high growth-promoting activity, can be utilized as a microbial inoculant to promote hairy vetch and soybean growth in sustainable agriculture.

## 1 Introduction

The application of agrochemicals in agriculture has significantly increased crop production. However, overdependence on these chemicals can have long-term detrimental effects on soil properties and functions, as well as greenhouse gas emissions (Prashar and Shah, 2016). To address this issue, various alternative approaches have been developed, such as the use of cover crops (Grismer, 2000), biofertilizers, and biological control agents (Jacoby et al., 2017), to minimize the overdependence on and negative impacts of agrochemicals.

Hairy vetch (*Vicia villosa* Roth subsp. *villosa*) is a widely used cover crop in Asia and Europe (Fujii, 2001). It is generally used as a pre-crop or cover crop in rotation systems to control weeds, suppress diseases, and improve soil structure and porosity (Kamo et al., 2003). As a legume, hairy vetch has the potential to add 20–25 kg N 10 a^–1^ to the soil, mainly through symbiosis with nitrogen-fixing bacteria (Abdollah et al., 2020; Kim et al., 2020). Several studies have demonstrated that the incorporation of hairy vetch significantly enhances fungal biomass and alters fungal community composition in the soil compared to the application of chemical fertilizers (Kataoka et al., 2017; Asghar and Kataoka, 2022).

Endophytes are microbes that reside within different plant tissues without causing apparent disease symptoms (Gupta et al., 2020). Their diversity and community structure are influenced by various factors, such as plant species, tissue type, and growth location (Aly et al., 2011; Gonzaga et al., 2015; Dastogeer et al., 2020). Plant–endophyte symbiotic associations can exist in different plant tissues, such as leaves, stems, and roots, and may persist throughout all or part of the plant life cycle (Poveda et al., 2022). Endophytic fungi provide several benefits to their host plants (Rim et al., 2021), such as protection against biotic (Billar de Almeida et al., 2020; Adeleke et al., 2022) and abiotic stresses (Lata et al., 2018; Batzer and Mueller 2020; Xiao et al., 2021). In addition, endophytes promote the growth of their host plants by enhancing the bioavailability of soil nutrients through solubilization activity (Baron and Rigobelo, 2022; Verma et al., 2022) and the production of various plant hormones (Porras-Alfaro et al., 2011; Gupta et al., 2020).

Although long overlooked, fungal endophytes have been isolated from a wide variety of plant species and are increasingly being recognized for their ecological significance, particularly their disease-suppressive and growth-promoting abilities (Costa et al., 2021). Many studies have revealed the composition and diversity of fungal communities in the rhizosphere of legumes, such as soybean, cowpea, and *Arabidopsis* (Zhang et al., 2019; Chang et al., 2021; Guo et al., 2022). However, relatively few studies have focused on the isolation and functional characterization of legume-associated fungal endophytes. Exploring the endophytic fungal communities of hairy vetch could advance our understanding of plant–endophyte associations and provide fungal strains with disease-suppressing and growth-promoting abilities that can be utilized in agriculture.

The present study aimed to assess the diversity of endophytic fungi associated with different hairy vetch tissues collected from eight different regions in Japan and to investigate the effects of soil properties and growth location on the community composition and structure of these fungi. Additionally, we evaluated the functional potential of endophytes associated with hairy vetch plants and how they contribute to the growth of hairy vetch and other legumes.

## 2 Materials and methods

### 2.1 Soil sampling

Hairy vetch plants were sampled from eight different fields across Japan where hairy vetch/soybean rotation is practiced (**Table 1**). Three representative plants were collected as replicates from each sampling site between March and June 2020. Bulk soil from the top 15 cm was sampled from each location for physicochemical analysis. The collected soil samples were air-dried at room temperature for 2 to 3 days and passed through a 2-mm sieve to remove all plant debris prior to analysis.

**Table 1:** Sampling locations and information on soil physicochemical properties of hairy vetch plants across Japan.

Sampling location	Latitude/longitude	Temperature Min~ Max	Annual precipitation (mm)	Cultivation history	Soil chemical property						
					pH	EC mS/m	TN (g kg^−1^)	(NH_4_–N) (mg kg^−1^)	STP (mg kg^−1^)	TK (mg kg^−1^)	SOM %
Akita (AK)	39°80′N/140°05′E	0°C–25°C	101.16mm	Soybean	6.91	3.8	0.63	0.092	0.076	64.0	>12.0
Ogata (OG)	40°1′N/139°57′E	0.2°C–24°C	202.4mm	Soybean	6.10	7	0.45	0.063	0.050	70.0	10.8
Fukushima (FK)	37°35′N/140°25′E	1°C–15°C	69.74mm	Soybean	5.43	5.2	0.30	0.051	0.056	32.9	9.8
Fuchu (FC)	35°68′N/139°47′E	2.8°C–30.4°C	35.51mm	Soybean	6.80	6.7	0.86	0.043	0.092	96.6	>12.0
Odawara (OD)	35°16′N/139°09′E	11.6°C–18.3°C	97.23mm	Soybean	6.71	7.3	0.41	0.029	3.023	38.9	8.3
Kanagawa (KN)	35°17′N/139°63′E	11.4°C–18.2°C	96.28mm	Soybean	7.22	6.5	0.58	0.050	0.037	46.0	>12.0
Inami (IN)	34°45N/134°55′E	14.2°C–18.6°C	57.44mm	Soybean	4.71	4.1	0.54	0.060	0.036	39.6	11.5
Himeji (HM)	34°49′N/134°41′E	12.0°C–18.2°C	98.83mm	Soybean	4.23	6.5	0.33	0.052	0.015	48.2	>12.0

EC, electrical conductivity; TN, total nitrogen; STP, soil total phosphorus; TK, total potassium; SOM, soil organic matter.

### 2.2 Soil physicochemical analysis

Soil pH and electrical conductivity (EC) were determined in 1 M KCl and deionized water at a soil-to-solution ratio of 1:5. The dry combustion method and an NC analyzer (VEGETECH Physical and Chemical Analysis Center, Kanagawa, Japan) were used to measure total carbon (TC) and total nitrogen (TN). Inorganic N was extracted from the soil using 2 M KCl, and the N–NH_4_^+^ content in the extract was analyzed using the modified indophenol blue method. The soil total phosphorus (STP) content was determined by perchloric acid digestion (Sharpley, 1996), and the total potassium (TK) content was measured via flame photometry using the Jackson method (Walkley and Armstrong Black, 1934). Soil organic matter (SOM) was estimated using the Walkley and Black volumetric method (Mohanram and Kumar, 2019).

### 2.3 Isolation of endophytic fungi from hairy vetch

Three hairy vetch plants from each location were used for the isolation of endophytic fungi. Plant tissues were cleaned and sterilized according to the protocol described in our previous study (Taheri et al., 2022). Briefly, the tissues were washed three times with distilled water, immersed in 70% ethanol for 20 s, and then immersed in 1% sodium hypochlorite for 20 s. The tissues were finally rinsed three times with sterilized distilled water and cut into small pieces (5 mm × 5 mm) using a sterile scalpel. Approximately six to eight root or leaf segments from each replicate plant from each location were placed in a Petri dish containing potato dextrose agar (PDA) supplemented with 50 μg mL^−1^ chloramphenicol. The Petri dishes were placed in an incubator (Sanyo MIR-253 Refrigerated Incubator) at 28°C ± 2°C under dark conditions and monitored regularly for fungal growth over a 15-day period. The hyphal tips that emerged were subsequently transferred to new PDA plates to obtain pure cultures for DNA extraction and identification. The colonization frequency (CF) of endophytic fungi was evaluated using the formula described by Petrini (1991).


$$\begin{array}{c} \text{CF }\left(\%\right)\;=\;(\text{number of segments showing endophyte growth}\\ /\text{number of segments plated})\times 100. \end{array}$$

### 2.4 Identification of endophytic fungi by ITS sequencing

The recovered fungi were grouped into different morphotypes based on culture characteristics such as the shape, size, color, and growth pattern of the fungal colonies. The total DNA of all representative morphotypes was extracted using a plant mini kit (QIAGEN, Hilden, Germany) following the manufacturer’s protocol. The nuclear ribosomal internal transcribed spacer (ITS) regions were amplified using a universal primer pair targeting ITS1 (5′ TCCGTAGGTGAACCTGCGG 3′) and ITS4 (5′ TCCTCCGCTTATTGATATGC 3′). PCR amplification was performed in 25-μL reactions containing 12.5 μL of Go Taq DNA polymerase (Promega, Madison, WI, USA), 2 μL of each primer, and 2 μL of extracted DNA template. The following modified conditions were used for amplification: initial denaturation at 95°C for 5 min; 35 cycles of denaturation at 95°C for 1 min, primer annealing at 54°C for 1 min, and extension at 72°C for 1 min; and final extension at 72°C for 5 min. The amplified ITS fragments were subsequently cleaned using a Nucleo-Spin gel and PCR clean-up kit (Macherey-Nagel, Düren, Germany) following the manufacturer’s instructions and subsequently sequenced. The ITS sequences of each isolate were analyzed using a BLAST search (blast.ncbi.nlm.nih.gov) to identify the fungal species. A threshold of ≥95% similarity was implemented to differentiate between taxa.

### 2.5 Community composition of endophytic fungi associated with hairy vetch

The α diversity of the endophyte species within the samples was determined using the Shannon–Wiener biodiversity index (H′) and the Chao1 estimator. Analysis of variance (ANOVA) was used to evaluate the diversity index values among locations and tissue types. To compare differences in community composition and structure (β diversity) among locations and tissue types, analysis of similarity (ANOSIM) and PERMANOVA were performed. The Bray–Curtis coefficient, which considers the abundance of taxa in addition to the presence or absence of particular fungal taxa, and the Jaccard index, which considers the presence or absence of fungal taxa among samples, were applied as two different pairwise similarity measures to provide different insights for comparing communities. PAST ver. 3.26 was utilized to analyze the α and β diversity.

### 2.6 Characterization of plant growth-promoting traits of fungal isolates

#### 2.6.1 Indole-3-acetic acid production

The ability of the fungal isolates to produce indole-3-acetic acid (IAA) was evaluated using the Salkowski reagent method (Patel et al., 2018). Briefly, three fungal plugs were inoculated into tubes containing 5 mL of yeast extract peptone dextrose (YPD) broth (per liter: 10 g yeast extract, 20 g peptone, and 20 g dextrose; final pH 6.0) and incubated at 26°C for 5 days on a rotary shaker at 180 rpm. The cultures were then centrifuged at 10,000 rpm for 10 min and filtered through a 0.2-μm filter (Millipore, Burlington, MA, USA). Filtered cultures (1 mL) were mixed with 1 mL of Salkowski reagent. The IAA concentration was determined from a calibration curve using standard chemicals.

#### 2.6.2 Phosphate and potassium solubilization activities

The phosphate- and potassium-solubilizing abilities of the isolates were evaluated on solid Pikovskaya medium supplemented with 0.5 g yeast extract, 10 g dextrose, 5 g calcium phosphate, 0.5 g ammonium sulfate, 0.2 g potassium chloride, and 0.1 g magnesium sulfate (per liter) (Doilom et al., 2020) and on Alexandrow medium supplemented with 5 g glucose, 0.5 g MgSO_4_.7H_2_0, 0.005 g FeCl_3_, 0.1 g CaCO_3_, 2 g Ca_3_(PO_4_)_2_, and 2 g sericite mica (per liter) (Meena et al., 2014). In triplicate, one fungal mycelial plug was transferred to Pikovskaya and Alexandrov agar plates for each isolate. PDA plugs were transferred onto similar plates as a control. The phosphate- and potassium-solubilizing activities of each isolate were assessed by measuring the diameter of the clearance zone around the colony.

#### 2.6.3 Siderophore production activities

The production of siderophores by the fungal isolates was evaluated qualitatively using the chrome azurol S (CAS) half-plate agar assay (Milagres et al., 1999) and quantitatively using the CAS liquid assay (Osman et al., 2019). The number of siderophore units was calculated using the following formula:


%Siderophores=[(Ar−As)/Ar]×100

where Ar is the reference absorbance at 630 nm (CAS reagent only) and AS is the sample absorbance at 630 nm.

### 2.7 Plant inoculation assay

Hairy vetch (*V. villosa* Roth subsp. *villosa*) and soybean (*Glycine max* (L.) Merr cv. Enrei) seeds were surface sterilized and pregerminated in moisture-saturated plastic Petri dishes for 24–48 h at 25°C. Germinated hairy vetch seeds were sown in 142-cm^2^ plastic pots filled with sterilized, fertilized, and granulated soil (Nippi No. 1, Nippon Hiryo, Tokyo, Japan). Germinated soybean seeds were planted in CUL-JAR300 plant boxes (Iwaki, Japan) filled with sterilized vermiculite. The seeds of hairy vetch and soybean were inoculated with 2 mL of the selected fungal spore suspension (10^7^ spores mL^−1^) on two occasions: at sowing (concentrically around each seed) and 1 week after planting (arranged concentrically around the shoot on the surface of the vermiculite and soil for both soybean and hairy vetch). Hairy vetch plants were grown in a phytotron at 26°C and watered with normal tap water. In contrast, the soybean plants were grown in a plant growth chamber maintained at 25°C and 70% relative humidity under a 16/8-h day/night cycle and watered with sufficient B&D nitrogen-free solutions (Broughton and Dilworth, 1971). Twenty days after the second inoculation, the shoot and root fresh weights and heights of both hairy vetch and soybean plants were measured. Data were collected from five plants in three independent experiments for both hairy vetch and soybean. Successful fungal colonization was evaluated by staining the hairy vetch and soybean roots according to the protocol described by Phillips and Hayman (1970).

### 2.8 Statistical analysis

Statistical analyses were performed using one-way ANOVA, followed by Tukey’s honestly significant difference (HSD) test with 5% probability.

## 3 Results

### 3.1 Soil chemical properties

The pH of the collected soils ranged from 4 to 7.50, with Kanagawa soil having the highest pH (7.22) and Himeji soil having the lowest (4.23). Odawara soil had the highest EC content (7.3 mS/m) and total phosphate content (3.23 mg/100 g), but the lowest organic matter content. The soil collected from Fuchu had slightly greater TN content than the other locations, whereas the soil sample collected from Akita had the highest content of ammonium nitrogen (NH_4_–N) (**Table 1**).

### 3.2 Diversity and abundance of recovered fungi associated with hairy vetch

**Figure 1:**
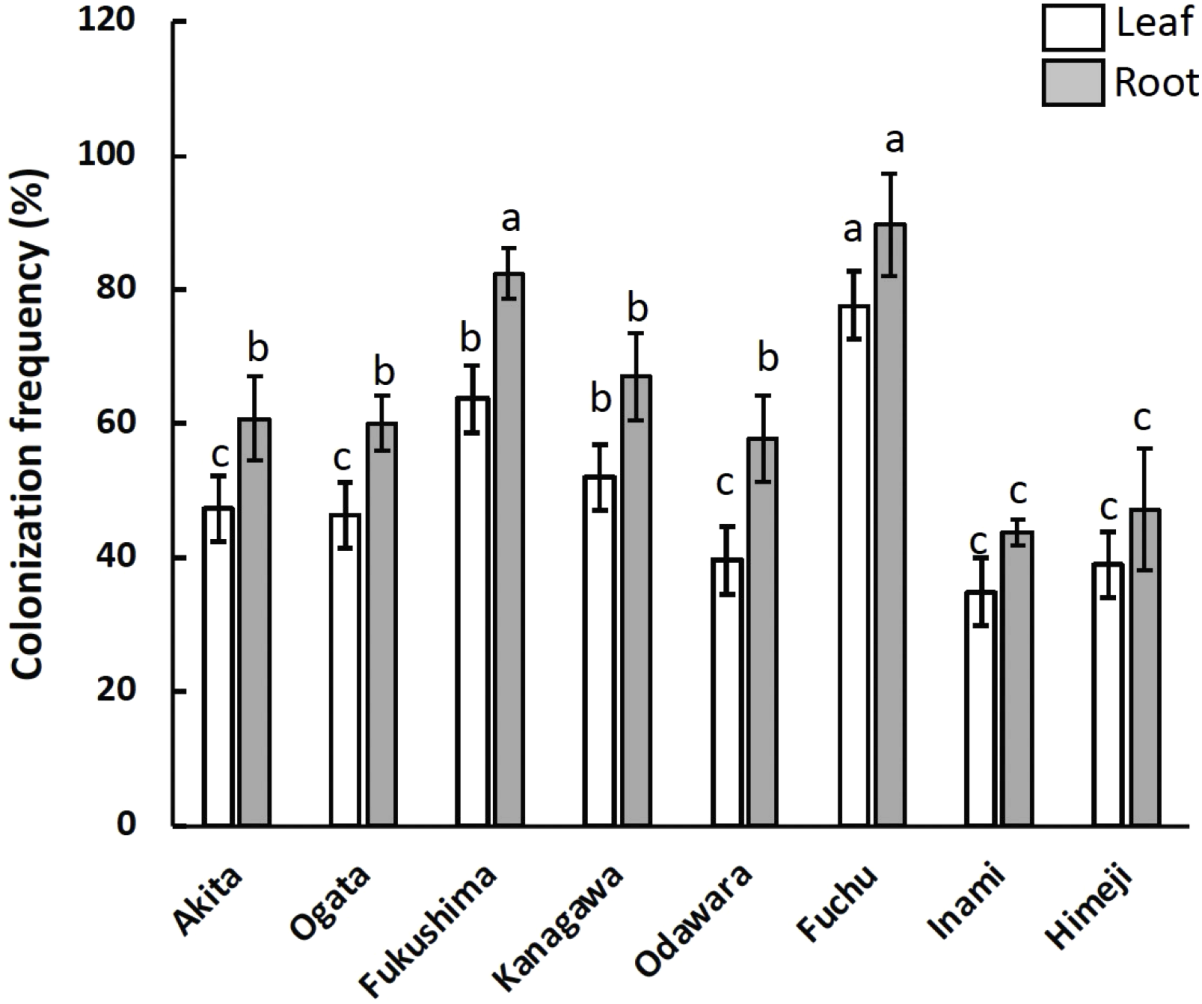
Colonization frequency (CF) of fungal endophytes associated with hairy vetch (*Vicia villosa*) collected from eight different locations and isolated from different tissues. The CF refers to the mean number of tissues colonized with fungal endophytes and is expressed as a percentage (%). The error bar indicates the standard error (SE) of the mean. P-values were obtained by ANOVA to indicate significant differences (*p* < 0.05).

**Figure 2:**
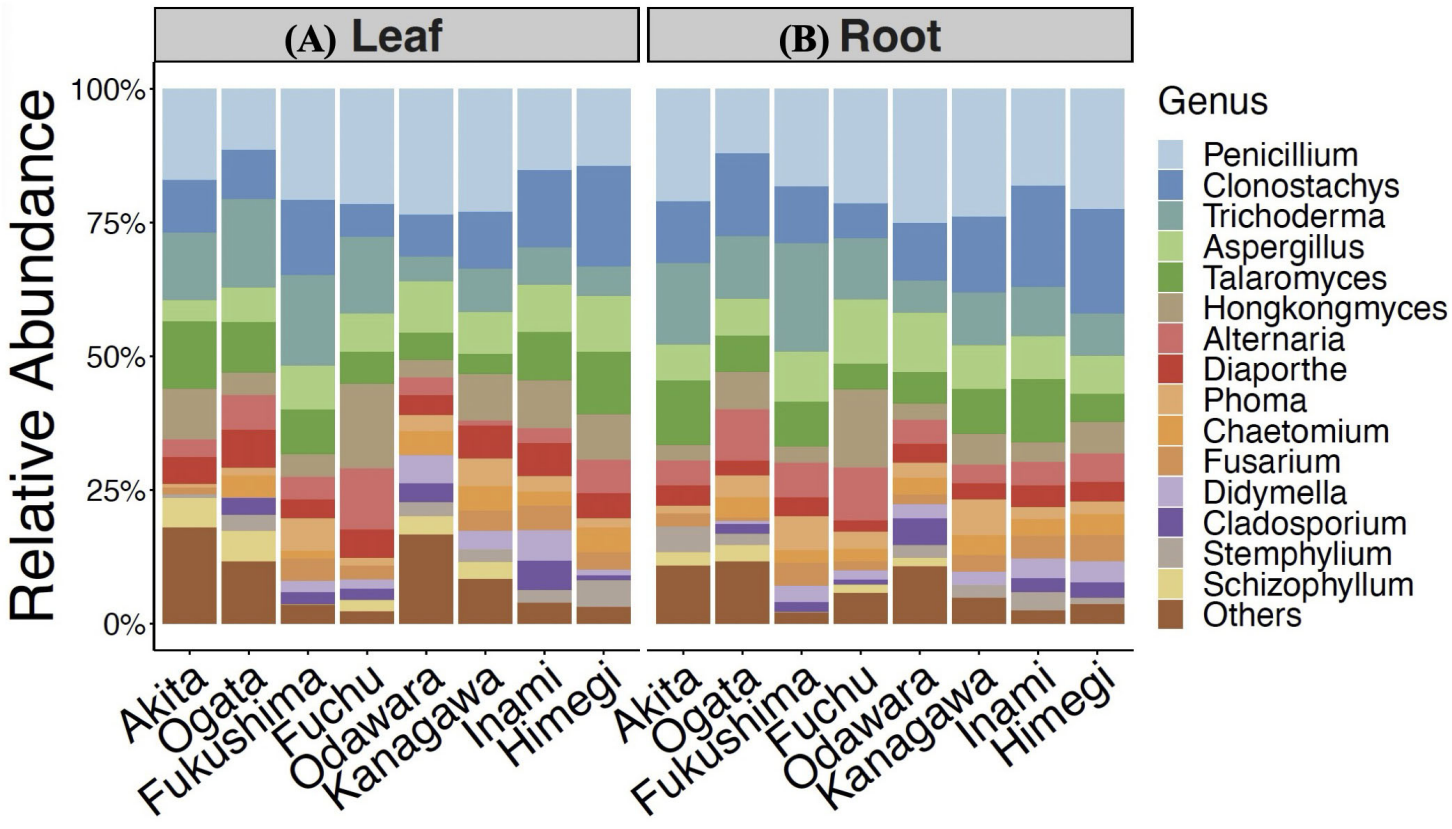
Relative abundance of endophytic fungi isolated from the leaves **(A)** and roots **(B)** of hairy vetch plants collected at different sampling locations.

A total of 2,186 fungal isolates were recovered from 9,600 segments of hairy vetch leaves and root tissues sampled from eight different locations. The colonization frequency differed significantly among the sampling locations and tissues (**Figure 1**). The highest colonization of fungal endophytes was observed in samples from Fuchu and Fukushima, with colonization rates of 91.5% and 84.1%, respectively (**Figure 1**). Root tissues were colonized more frequently than leaves at all locations (**Figure 1**). Clustering of operational taxonomic units (OTUs) based on ITS sequencing revealed 80 OTUs belonging to three phyla, five classes, 10 orders, and 69 genera of fungi. The sequences were deposited in the National Center for Biotechnology Information (NCBI) GenBank and compared with those already deposited via BLAST searches (**Supplementary Table S1**). *Penicillium*, *Clonostachys*, *Trichoderma*, *Aspergillus*, *Talaromyces*, *Hongkongmyces*, *Fusarium*, and *Alternaria* were the most abundant genera in all the samples; however, their distributions varied based on the sampling location and tissue (**Figure 2**). All 15 of the most dominant fungal genera in this study were isolated from both leaf and root tissues collected from Fuchu, Ogata, and Odawara. In contrast, in other locations, including Kanagawa, not all fungal genera were isolated from both leaf and root tissues. For instance, *Schizophyllum* was isolated from leaf tissues but not from the roots in Kanagawa. Furthermore, *Schizophyllum* was not isolated from Himeji and Inami, indicating that the sampling location influences the fungal community associated with hairy vetch (**Figure 2**).

**Figure 3:**
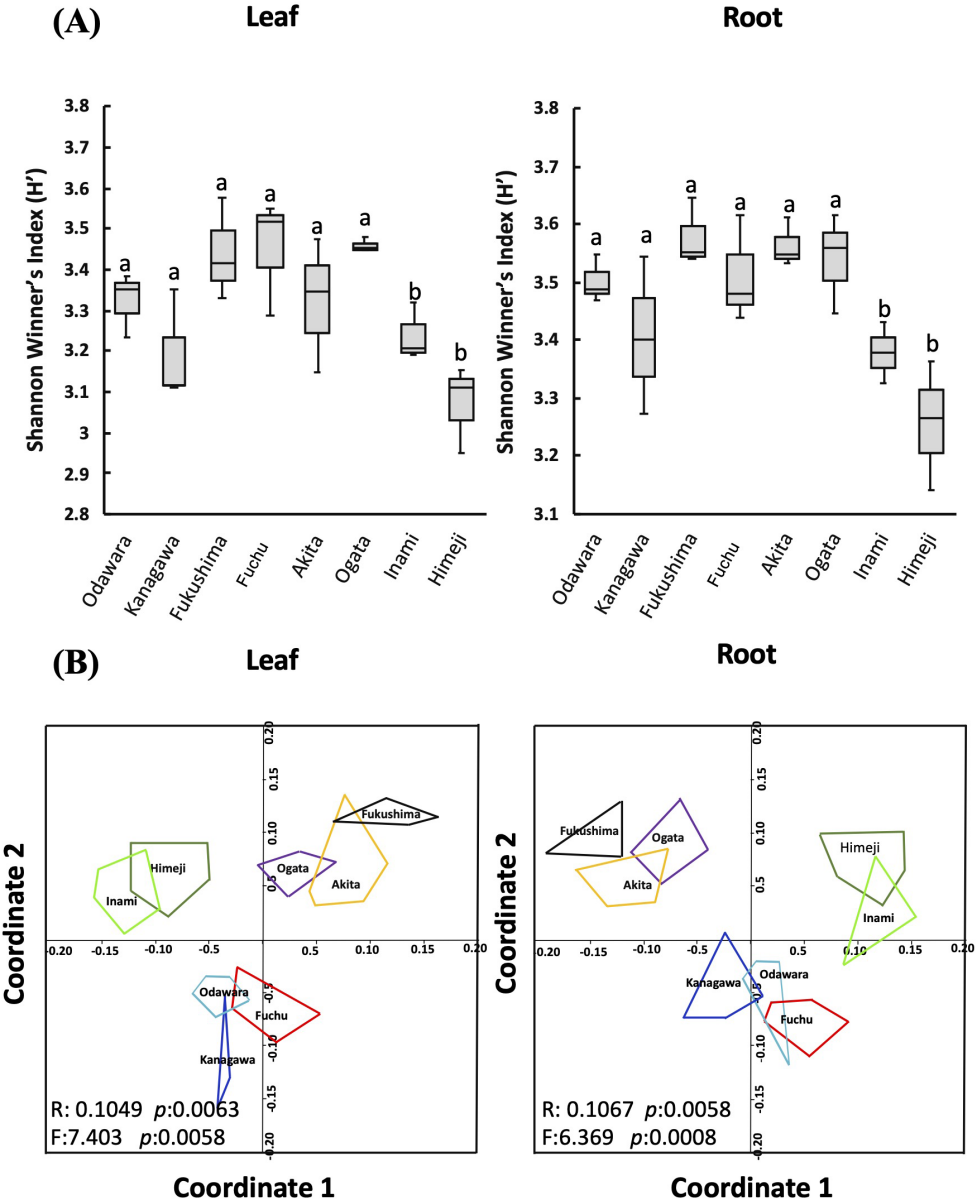
Fungal endophyte biodiversity analysis. The effect of different host tissues (leaf and root) and sampling locations on fungal endophyte biodiversity as measured by **(A)** the Shannon-Wiener biodiversity index (H′). Within each figure, different letters (a, b) above the bars indicate significant differences (*p* < 0.05, ANOVA and Tukey’s HSD tests). **(B)** Non-metric multidimensional scaling (NMDS) plots for cluster analysis of the fungal endophyte community were calculated with the Bray–Curtis coefficient. The ANOSIM statistic R and the PERMANOVA statistic F values and the corresponding *p* values indicating the significance of dissimilarity obtained by permutation of group membership, with 9,999 replicates.

The Shannon–Wiener biodiversity index showed significant variation among the tissues at different sampling locations, with samples from Inami (H′ = 3.30) and Himeji (H′ = 3.16) in Hyogo Prefecture having the lowest diversity. Root samples had greater richness than leaf samples at all locations (**Figure 3A**). Similarly, univariate analysis revealed that microbial diversity (Shannon index) (**Figure 3A**) and abundance (Chao1) (**Supplementary Figure S1A**) significantly differed (*p* < 0.05) among locations and tissues.

Ordination analysis was performed to investigate the distance between the community composition of fungal endophytes isolated from the leaf and root tissues of hairy vetch collected from different sampling locations (**Figure 3**; **Supplementary Figure S1B**). Two-dimensional non-metric multidimensional scaling (NMDS), ANOSIM, and PERMANOVA, which are based on the clustering of fungal communities, revealed that sampling location had a significant impact on the structure and assembly of endophyte communities. The results showed that endophytic fungi isolated from leaf and root tissues of plants from Himeji and Inami have similar fungal community compositions and were clustered together. Similarly, endophytic fungi isolated from Akita, Ogata, and Fukushima clustered together, whereas endophytes isolated from Fuchu, Kanagawa, and Odawara showed greater similarity and clustered together (**Figure 3B**). These results suggest that location plays a crucial role in determining the composition and structure of endophytic fungal communities in plants (**Figure 3**; **Supplementary Figure S1B**). Moreover, this study revealed that plants collected from distant locations did not have similar endophytic fungal community compositions. These findings highlight the importance of location and environmental factors in the diversity and distribution of endophytic fungi in plant tissues.

### 3.3 Correlations between soil properties and fungal communities

**Figure 4:**
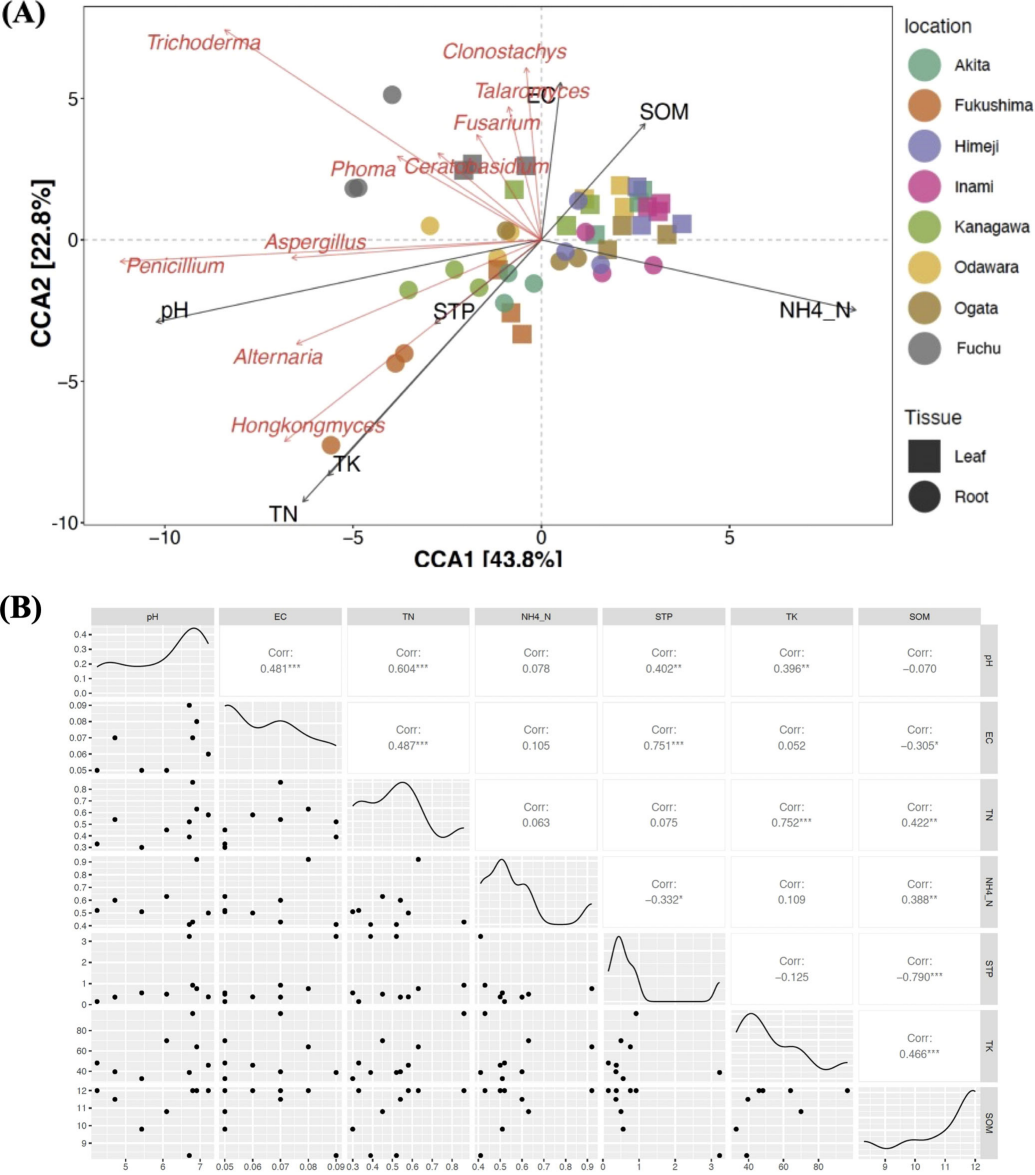
**(A)** Canonical correspondence analysis (CCA). Correlations between the most abundant genera and soil physicochemical characteristics. A longer line indicates a greater effect of the feature on the fungal distribution. The angle and relative direction between the soil features and the fungal genera indicate their correlation type. The green lines represent soil factors, the black dots indicate sampling locations, and the blue dots indicate fungal genera. The CCA1 and CCA2 axes explained 58.26% and 14.52% of the observed variance, respectively. **(B)** Pearson’s correlation analyses of the soil properties at eight different locations. Abbreviations: SOM, soil organic matter; TN, soil total nitrogen; STP, soil total carbon; TC, total carbon; TK, total potassium; NH_4_–N, ammonium content; EC, electrical conductivity.

Canonical correspondence analysis (CCA) revealed the effects of soil properties on fungal community composition. The first and second CCA axes explained 43.8% and 22.8% of the variance, respectively, based on the 10 most abundant fungal genera at each location (**Figure 4A**).

SOM and NH_4_–N significantly influenced the abundance of fungi such as *Trichoderma* and *Clonostachys*. In contrast, pH inversely affects *Penicillium* abundance. Samples from Akita and Fukushima (green and orange) are distributed along the negative axes of CCA1 and CCA2, indicating a lower influence of pH and a greater influence of TN/TK. However, samples from regions such as Ogata and Fuchu (brown and gray) are clustered around high-SOM and high-NH4–N areas. Pearson’s correlation analysis revealed a significant positive correlation between soil pH and STP, TN, and TK contents, whereas a significant negative correlation was found between soil pH and SOM content (**Figure 4B**).

### 3.4 Plant growth-promoting traits of all isolates

The plant growth-promoting (PGP) traits of the isolates, including IAA synthesis, phosphate (P) and potassium (K) solubilization, and siderophore production, were assessed (**Supplementary Table S3**). Of the 80 identified isolates, 42 exhibited the potential to produce IAA. *Penicillium* showed the highest IAA production, with FCR2 (*Penicillium simplicissimum*) isolated from root tissue in Fuchu and AKL25 (*Penicillium griseofulvum*) isolated from leaf tissue in Akita having the highest potential (15.1 and 10.2 mg L^−1^, respectively). In terms of phosphate solubilization activity, 10 isolates formed clearance zones around their colonies (**Supplementary Table S3**; **Supplementary Figure S2**), with clearance zone sizes ranging from 1.10 to 2.20 cm among the isolates. AKR1 (*Alternaria* sp.), ODR41 (*Fusarium* sp.), and FCL18 (*Trichoderma hamatum*) showed high P solubilization potential, with AKR1 showing the widest clearance zone with a diameter of 2.20 cm. Of the 80 isolated fungi, 13 demonstrated K solubilization potential. FCR28 (*Trichoderma atrobrunneum*) showed the greatest potential to solubilize potassium, with a clearance zone size of 7.92 cm (**Supplementary Table S3**; **Supplementary Figure S3**), whereas the clearance zone size varied between 1.51 and 7.92 cm among the other isolates. Regarding siderophore production, 39 isolates were found to produce siderophores (**Supplementary Table S3**, **Supplementary Figure S4**). AKL25 (*P. griseofulvum*) and FCR28 (*T. atrobrunneum*) produced the highest siderophore concentrations. Moreover, the majority of the *Trichoderma* species isolated in the present study showed the potential to produce high amounts of siderophores. Of the 80 tested isolates, 66 showed at least one PGP trait. Two isolates (AKR9 and FCR10) exhibited all four traits, six isolates exhibited three PGP traits, and the remaining isolates exhibited two or fewer growth potential traits.

### 3.5 Effects of fungal inoculation on the growth of hairy vetch and soybean

**Figure 5:**
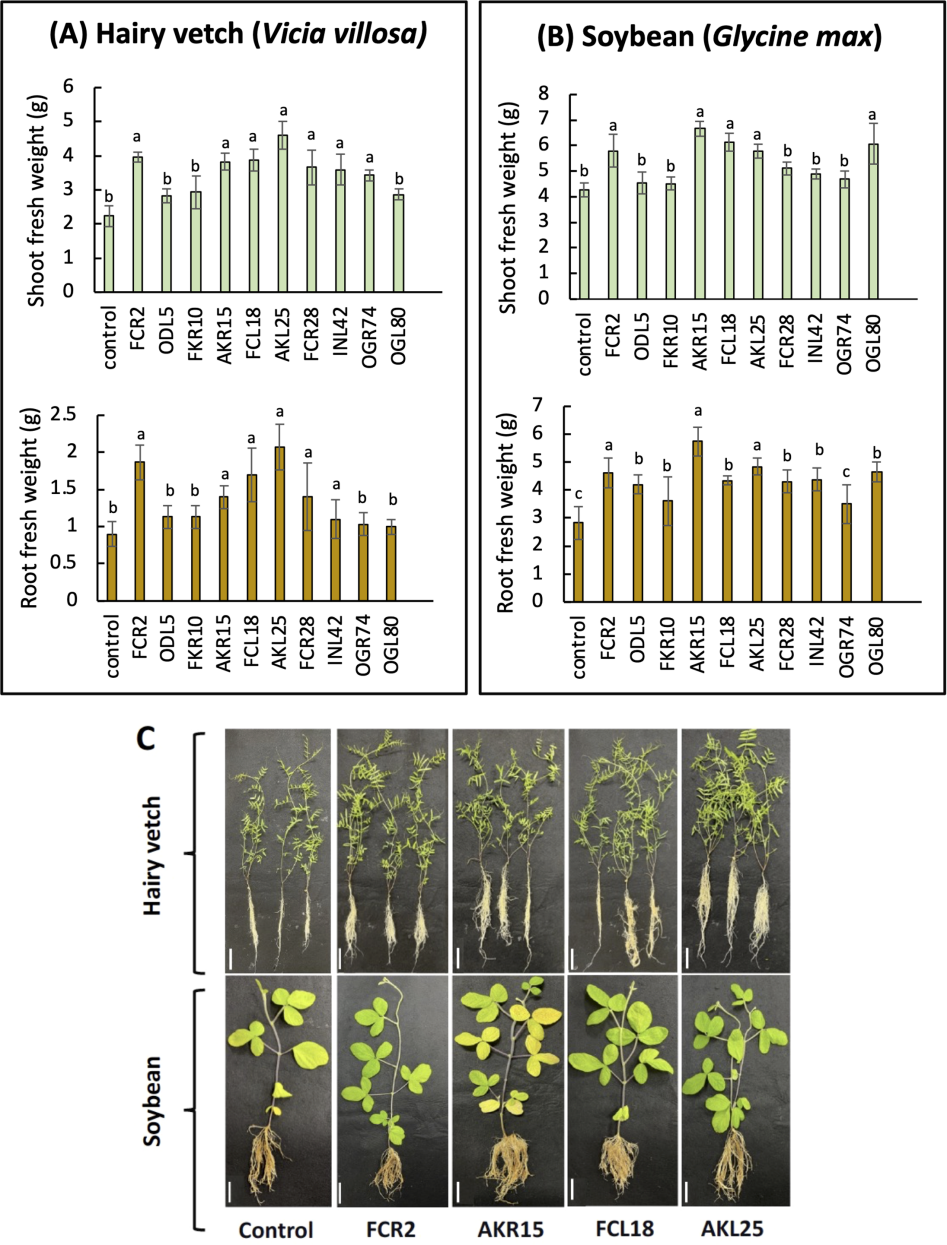
The influence of endophytic fungi on **(A)** the shoot and root fresh weights of hairy vetch (*Vicia villosa*) and **(B)** soybean (*Glycine max*) and **(C)** visualization of inoculated plants after 20 days (scale bar, 5 cm). Differences were considered significantly different at *p* < 0.05 according to one-way ANOVA with Tukey’s test (n = 5); means ± SDs. For each inoculation treatment, bars with different letters (a, b) indicate that the mean values are significantly different at a level of *p* < 0.05.

Based on the results of the PGP characterization, 10 isolates that showed at least two PGP traits with high values were selected for plant inoculation tests (**Table 2**). Compared with those of the control plants, the shoot and root fresh weights of the plants inoculated with six isolates (FCR2, AKR15, FCL18, AKL25, FCR28, and INL42) were significantly higher (**Figure 5A**). Among the six isolates, AKL25 (*P. griseofulvum*) yielded the highest increase in root and shoot weight and volume. Notably, inoculation with OGR74 promoted shoot growth, whereas inoculation with ODL5, FKR10, or OGL80 did not significantly change the shoot or root fresh weight of hairy vetch plants (**Supplementary Figure S5A**).

**Table 2:** Selected isolates for plant inoculation assay.

	Species	Origin of isolate	Tissue	IAA	Phosphate solubilization	Potassium solubilization	Siderophore production
FCR2	*Penicillium simplicissimum*	Fuchu	Root	15.1 ± 0.13	0	0	37.0 ± 0.12
ODL5	*Stemphylium lycopersici*	Odawara	Leaf	8.00 ± 0.11	0	2.85 ± 0.03	39.4 ± 0.07
FKR10	*Trichoderma koningiopsis*	Fukushima	Root	9.55 ± 0.04	1.16 ± 0.05	4.24 ± 0.02	0
AKR15	*Trichoderma koningii*	Akita	Root	6.23 ± 0.03	1.25 ± 0.02	4.26 ± 0.06	61.0 ± 0.04
FCL18	*Trichoderma hamatum*	Fukushima	Leaf	6.11 ± 0.10	1.27 ± 0.02	0	60.3 ± 0.10
AKL25	*Penicillium griseofulvum*	Akita	Leaf	10.2 ± 0.23	0	0	64.7 ± 0.07
FCR28	*Trichoderma atrobrunneum*	Fuchu	Root	1.35 ± 0.05	1.21 ± 0.06	7.92 ± 0.03	63.8 ± 0.03
INL42	*Phoma* sp.	Inami	Leaf	5.80 ± 0.03	0	0	33.0 ± 0.02
OGR74	*Aspergillus nomiae*	Ogata	Root	5.06 ± 0.04	0	1.51 ± 0.05	0
OGL80	*Alternaria alternata*	Odawara	Root	2.70 ± 0.07	0	2.11 ± 0.01	0

IAA: observed based on the formation of pink color and by checking the absorbance at 530 nm with a microplate reader and equal to mg L^−1^. Phosphate solubilization: The unit represents the size of the halo zone (cm) caused by the dissolution of tricalcium phosphate. Potassium solubilization: The unit represents the size of the halo zone (cm) caused by the dissolution of potassium minerals (sericite mica). Siderophore production: unit represents the size of the yellow zone (cm).

FC, Fuchu; OD, Odawara; FK, Fukushima; AK, Akita; IN, Inami; OG, Ogata; L, leaf; R, root.

Furthermore, five isolates (FCR2, AKR15, FCL18, AKL25, and OGL80) increased soybean shoot and root fresh weights, of which AKR15 showed the most robust growth promotion (**Figure 5B**), whereas inoculation with OGR74 did not affect the growth of soybean plants.

Of the 10 endophytes selected, four isolates (FCR2, AKR15, FCL18, and AKL25) exhibited greater growth-promoting effects on both hairy vetch and soybean plants compared to the control (**Figure 5C**). AKL25 and AKR15 had the greatest ability to promote shoot and root growth, respectively, in hairy vetch and soybean plants. Conversely, OGR74 and OGL80 exhibited the potential to enhance only hairy vetch and soybean growth, respectively. These results indicate the specificity of the isolated endophytic fungi in their ability to promote plant growth (**Figure 5**; **Supplementary Figure S5B**). Notably, inoculation with any of the isolates did not cause any negative effects or disease symptoms in either hairy vetch or soybean plants.

**Figure 6:**
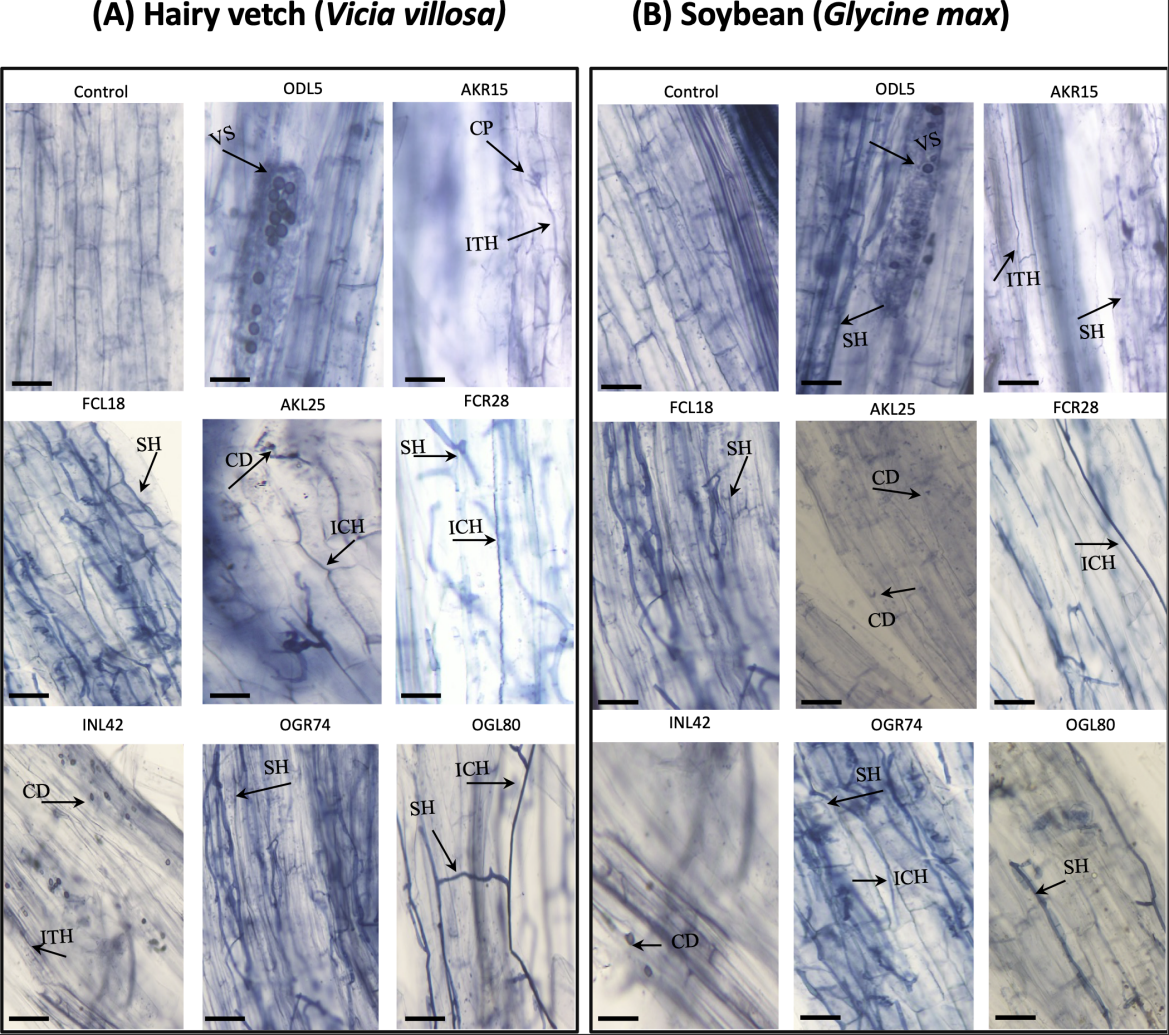
Endophytic fungi inhabiting longitudinal sections of planted **(A)** hairy vetch (*Vicia villosa*) and **(B)** soybean (*Glycine max*) plants observed under light microscopy of non-inoculated and inoculated root tissues. Distribution of vesicles in the root cortex (EN5); intracellular colonization (EN25 and EN79) and intercellular colonization (EN15) of endophytic fungi; growth of conidia (EN42); penetration of septate hyphae (EN18 and EN73); and formation of conidiophores (EN25) in the roots of hairy vetch and soybean. The arrowhead shows the fungal structures, which are indicated as follows: vesicles (VS), conidiophores (CP), septate hyphae (SH), conidia (CD), intercellular hyphae (ICH), and intracellular hyphae (ITH).

The roots of both hairy vetch and soybean plants inoculated with fungal isolates were examined to investigate fungal colonization. Upon inoculation with the selected fungi, typical fungal structures (septate hyphae, conidia, vesicles, and conidiophores) were observed within the root tissues of both plant species. The majority of fungal isolates showed the potential to penetrate the intercellular or intracellular spaces of the roots of both hairy vetch and soybean plants, with AKR15 (*Trichoderma koningii*), AKL25 (*P. griseofulvum*), FCR28 (*T. atrobrunneum*), and OGR74 (*Aspergillus nomiae*) displaying intercellular penetration, and ODL5 (*Stemphylium lycopersici*), FCL18 (*T. hamatum*), INL42 (*Phoma* sp.), and OGL80 (*Alternaria alternata*) colonizing the intracellular spaces of both plant species (**Figure 6**). In general, the root tissues exhibited fungal hyphae colonizing the intercellular spaces of the roots, although only a few stained roots exhibited fungal vesicles, conidia, and conidiophores. The colonization of INL42 (*Phoma* sp.) was greater in the roots of hairy vetch than in the roots of soybean plants, indicating the preference of some isolates for colonizing specific plant roots. In the case of AKL25, FCR28, OGR74, and OGL80, dark and blue hyphae grew along the epidermis or cortex, parallel to the longitudinal axis of the roots (**Figure 6**).

## 4 Discussion

Although the functional potential of hairy vetch is well studied and its importance as a weed- and disease-suppressive species is well known (Yildirim et al., 2022), the interactions of endophytic fungi associated with hairy vetch and the effects of soil and environmental factors on the fungal community are poorly understood. In the present study, we analyzed the diversity of endophytic fungi associated with hairy vetch at various locations in Japan and the effects of soil properties, sampling location, and different tissues on the community and structure of endophytic fungi. Furthermore, the potential of the isolated endophytic fungi to promote the growth of hairy vetch and soybean plants was evaluated.

### 4.1 Community composition of endophytic fungi associated with hairy vetch

The present study revealed the diversity of endophytic fungi within hairy vetch at various locations, encompassing a broad taxonomic spectrum that includes three phyla, six classes, 13 orders, 22 families, 28 genera, and 80 species. Our findings are consistent with those of previous research, demonstrating that Ascomycota and Basidiomycota are the predominant fungal groups in both root and leaf samples. This alignment with established studies (e.g., Dastogeer et al., 2018; Khalil et al., 2021) reinforces the notion that these fungal groups are ubiquitous in many plant species due to their beneficial symbiotic relationships with plants and their adaptability. These fungi establish mutually beneficial relationships, aiding plants in the absorption of essential nutrients such as phosphorus while receiving carbohydrates in return. Their ability to produce diverse spores enables effective colonization of plant tissues. Furthermore, their tolerance to a wide range of environmental conditions ensures their thriving in various ecosystems, making them vital partners in the health and growth of many plant species.

Some genera of endophytic fungi were notably absent in some specific sampling locations (Fukushima, Inami, Himeji, and Kanagawa) with either highly acidic or alkaline soil pH (**Figure 2**). This finding indicates that some fungal genera are sensitive to low or high soil pH. Recent studies have reported a strong correlation between soil chemistry and soil microbial communities (Chen et al., 2021; Kang et al., 2021; Scarlett et al., 2021). Previous studies have also reported a significant relationship between soil pH and soil microbial community composition and structure, as specific fungi are unable to thrive in environments with very low or high soil pH (Lauber et al., 2009; Wang et al., 2015; Zhang et al., 2016; Liu et al., 2018; Noor et al., 2021). In the present study, the genera *Gibberella*, *Termitomyces*, and *Phomopsis* were not detected in tissues from locations with low soil pH, suggesting their sensitivity to pH differences (Ramrakhiani and Khowala, 2012). Moreover, low soil pH can increase the mobility of metal ions, causing heavy metal pollution that is detrimental to some fungal species (Kicińska et al., 2022).

The occurrence of endophytic fungi varies between various plant species and within diverse tissues of an individual plant (Yan et al., 2022) due to specific preferences exhibited by the colonizing fungi toward the host plant. In the present study, α diversity analysis revealed a more diverse endophytic fungal community in the roots of hairy vetch than in the leaves (*p* < 0.05). Furthermore, the true abundance of *Penicillium* was greater in root tissues at all sampling locations. *Penicillium* species are ubiquitous, which has been attributed to their lenient nutritional requirements and ability to grow over a wide range of conditions (Toghueo and Boyom, 2020). The findings of the present study are consistent with the dynamic distribution of endophytes, as the diversity of endophytes decreases from the lower to the upper part of the plant (Rosenblueth and Martínez-Romero, 2004). The diversity of endophytic fungi in roots is generally greater than that in shoots for several reasons, including the anatomical and physiological characteristics of plant roots, which provide more suitable microenvironments for fungal growth and colonization. The complexity of plant roots creates multiple niches for endophytic fungi. The roots of the majority of plants have several distinct zones, such as the root cap, meristem, elongation, and differentiation zones, each with its own unique physicochemical environment that can support the growth of different types of fungi. Moreover, the root system interacts with soil microorganisms, which can influence the composition of endophytic fungal communities in roots. Furthermore, the root system is involved in a range of physiological processes that attract endophytic fungi. For example, roots release a variety of exudates, such as sugars, amino acids, and organic acids, which can act as nutrient sources for fungi. Additionally, roots produce a range of secondary metabolites, such as flavonoids and terpenoids, which can serve as chemical signals to attract or repel endophytic fungi (Chen et al., 2020). However, in leaf tissues, microorganisms initially establish themselves on the surface of plant leaves through stomata, water holes, and wounds via horizontal transmission before eventually penetrating the leaf tissue.

### 4.2 PGP traits of hairy vetch endophytic fungi

Plant growth-promoting fungi (PGPFs) have been shown to enhance plant growth through various mechanisms, including facilitating the acquisition of essential elements such as nitrogen, phosphorus, and potassium, in addition to regulating phytohormone levels. Furthermore, PGPFs can promote growth directly through the production of IAA, the primary auxin in plants that regulates multiple growth and developmental processes (Teale et al., 2006). In the present study, the majority of the fungi evaluated exhibited the potential to produce IAA at different concentrations. Different studies have suggested that *Penicillium* species may produce greater amounts of IAA than other fungal species (Ikram et al., 2018; Toghueo and Boyom, 2020). Endophytic fungi isolated from various plant tissues have been reported to produce IAA; however, the potential for IAA production varies between isolates and plant tissues (Kumla et al., 2020). Several studies have reported that endophytic fungi isolated from plant roots have a high potential to produce IAA (Mehmood et al., 2019). Kumari et al. (2021) isolated endophytic fungi from the roots of *Azadirachta indica* and reported that some isolates produced high levels of IAA (Kumari et al., 2021). In the present study, *Penicillium* species produced a large amount of IAA. FCR2 (*P. simplicissimum*), which was isolated from roots, exhibited the greatest potential for IAA production at a concentration of 15.1 mg/L (**Supplementary Table S3**). Intriguingly, in the present study, the endophytic fungi isolated from leaf tissue, AKL11 and AKL25, which were identified as *Penicillium* species, also produced large amounts of IAA (12.5 and 10.2 mg/L, respectively). Overall, it can be concluded that endophytic fungi that are isolated from various plant tissues have the potential to produce IAA. However, the production potential may differ among different isolates and plant species.

The considerable amount of phosphorus applied to the soil quickly becomes insoluble, leading to the need for an alternative approach to prevent the excessive use of phosphatic fertilizers. One potential solution is to utilize endophytic fungi that can solubilize phosphate (Elias et al., 2016). Currently, researchers have identified approximately 60 species of fungi, primarily belonging to the genera *Alternaria*, *Aspergillus*, *Fusarium*, *Penicillium*, *Talaromyces*, and *Trichoderma*, with insoluble phosphate solubilization potential (Doilom et al., 2020). A literature review suggested that *A. alternata* is the most effective strain in terms of tricalcium phosphate solubilization, with a clearance zone of 2.6 cm (Ceci et al., 2018). This study also showed that fungal endophytes associated with hairy vetch can dissolve tricalcium phosphate in a solid medium. Among all the isolates tested in this study, AKR1 (*Alternaria* sp.) demonstrated a high solubilization potential for tricalcium phosphate (2.20-cm zone size), similar to a previous report (Ceci et al., 2018).

Siderophores, secondary metabolites with a high affinity for ferric iron, play a crucial role in iron uptake by plant roots, particularly under conditions of iron deficiency. These compounds effectively chelate metal ions, enhancing the bioavailability of iron to plants. Additionally, siderophores may indirectly promote plant growth by limiting the availability of iron to phytopathogens, thereby inhibiting their proliferation (Saha et al., 2016). Numerous fungi, including species of *Trichoderma*, produce various types of siderophores (Pecoraro et al., 2022). The production of siderophores benefits both plants and microorganisms in two significant ways: first, by restricting the growth of plant pathogens through iron deprivation, and second, by mobilizing previously inaccessible iron for plant uptake.

Our study demonstrated that the majority of fungi found in hairy vetch tissues are capable of synthesizing siderophores. Notably, *Trichoderma* species exhibited the highest potential for siderophore production. This high potential is attributed to their diverse and robust mechanisms for siderophore synthesis, which are often linked to their competitive nature and ability to thrive in various environments. Specifically, *T. atrobrunneum* (FCR28), *T. koningii* (AKR15), and *T. hamatum* (FCL18) were identified as having the highest siderophore production potential. These *Trichoderma* species are recognized for their effective iron-chelating capability (Trutmann and Keane, 1990), which enhances their competitive advantage over other microorganisms and contributes significantly to soil health and plant growth.

Moreover, *Trichoderma* species are commonly used in agriculture and have demonstrated great success in enhancing plant growth. This success can be attributed to the various mechanisms activated by *Trichoderma* species. These mechanisms primarily involve a reduction in plant disease severity and their effectiveness has been well documented (Subramaniam et al., 2022). AKR15 (*T. koningii*) and FCR28 (*T. atrobrunneum*) exhibited all four PGP traits. Various studies have focused on the potential of *T. koningii* as a biocontrol agent to suppress various plant diseases and enhance plant tolerance to biotic stress (Subramaniam et al., 2022). However, this is the first time that this species of *Trichoderma* has been isolated from hairy vetch plants with growth-promoting traits.

### 4.3 Plant growth promotion by hairy vetch endophytes

In the present study, we assessed the PGP activities of 10 fungal isolates based on their plant growth traits. After inoculation with the selected fungi, the growth parameters of the hairy vetch and soybean plants increased significantly (**Figure 5**). Kataoka et al. (2017) reported that the incorporation of hairy vetch into the soil significantly improves the soil fungal microbiome. These fungi are likely to remain in the soil and be transmitted horizontally from the soil to subsequent crops. The results of this study indicated that among all the tested endophytes, four isolates (FCR2, AKR15, FCL18, and AKL25) produce high amounts of IAA and siderophores and therefore show potential to promote the growth of both hairy vetch and soybean (**Figure 5**). Several studies have reported a positive correlation between IAA and siderophore production by endophytic fungi in promoting plant growth. A previous study reported that the endophytic fungus STL3G74 (*Aspergillus niger*) produces both IAA and siderophores and significantly promotes the growth of *Lolium perenne* (Tang et al., 2023). The correlation between the IAA and siderophore production potential of endophytic fungi and their ability to promote plant growth after inoculation has been well established in several studies, indicating that these traits are important for endophytic fungi to establish mutually beneficial relationships with their host plants. Although the mechanism underlying the positive correlation between IAA and the siderophore production potential of endophytic fungi and plant growth promotion is not fully understood, it has been suggested that IAA and siderophores may act synergistically to enhance plant growth by stimulating root development and nutrient uptake. Among the various isolates evaluated, AKL25, which was identified as *P. griseofulvum*, was the most prominent fungus promoting the growth of hairy vetch, whereas AKR15 (*T. koningii*) was the most effective in promoting the growth of soybean. Interestingly, OGL80 promoted soybean growth but not hairy vetch growth, whereas OGR74 promoted the growth of only hairy vetch. Endophytic fungi are known to promote plant growth via several mechanisms. However, not all endophytic fungi have the same effect on plant growth, and some may have different effects on shoot and root growth (Devi et al., 2023). The present study demonstrated the host specificity of fungal endophytes, which may exhibit different potentials based on their host plants.

In the present study, we isolated AKL25, which shares a high similarity with *P. griseofulvum* previously isolated from rice straw and exhibits a high potential for straw degradation (Guan et al., 2021). Interestingly, this strain has not been found to be associated with Leguminosae plants in the literature. Thus, for the first time, this species of *Penicillium* with a high potential to promote host plant growth has been isolated. Endophytic fungi are known to produce compounds that promote plant growth and development. For instance, the pathogenic fungus *Fusarium oxysporum* has been shown to synthesize volatile compounds that augment shoot and root growth in *Arabidopsis thaliana* and tobacco (Bitas et al., 2015). According to the results of the present study, AKL25 has the potential to produce high amounts of IAA and siderophores and to promote the growth of both soybean and hairy vetch. Thus, this isolate could be used as a potential bioinoculant to promote the growth of hairy vetch and soybean. Additionally, certain endophytic fungi can induce systemic resistance, which is a plant defense mechanism triggered in response to infection by pathogenic microorganisms (Morita et al., 2023). This mechanism provides protection against future pathogen attacks and enhances plant growth and development. In the present study, AKR15, which is highly similar to *T. koningii*, significantly promoted soybean growth. This species of *Trichoderma* has been previously identified as a biocontrol agent for plant diseases (Cutler et al., 1989; Rabinal and Bhat, 2020; Tripathi et al., 2021). The association of endophytic fungi with PGP traits may synergistically increase the growth of hairy vetch.

Microscopic observation of hairy vetch and soybean roots confirmed the presence of fungal structures. *Trichoderma* and *Penicillium* were the primary endophytic fungi used in the inoculation tests. *Trichoderma* species (FKR10, FCL18, and FCR28) are filamentous fungi that exhibit branched and septate hyphal structures, meaning that the fungus is composed of long, thin filaments that branch out and are separated by cell walls called septa. These structures allow *Trichoderma* to penetrate and colonize plant tissues. *Penicillium* species (AKL25), however, is also a filamentous fungus, but has a more compact structure. It produces a mycelium made of long, intertwined hyphae, which allows it to grow in a dense mass within plant tissues. *Penicillium* also produces spores that can be dispersed through the air and colonize new plant hosts. The hyphae of endophytic fungi can either exist intercellularly, such as in the case of AKR15 (*T. koningii*), AKL25 (*P. griseofulvum*), FCR28 (*T. atrobrunneum*), and OGR74 (*A. nomiae*), or exist intracellularly in plant tissues, such as in the case of ODL5 (*S. lycopersici*), FCL18 (*T. hamatum*), INL42 (*Phoma* sp.), and OGL80 (*A. alternata*), depending on the fungal species and host plant (**Figure 6**). Intercellular hyphae grow within plant cells, whereas intracellular hyphae grow inside plant cells (Khare et al., 2018). The mechanism underlying the intercellular or intracellular growth of endophytic fungi is not clearly understood, but it may be associated with fungal–host interactions and strategies used by fungi to colonize plant tissues. Some endophytic fungi secrete enzymes and other molecules that can degrade plant cell walls, enabling them to grow inside plant cells. Others are unable to penetrate plant cells and instead grow intercellularly, forming a network of hyphae that surround plant cells (Zhao et al., 2020). The ability of endophytic fungi to grow intracellularly or intercellularly can have different effects on the host plant. Intracellular endophytic fungi can provide nutrients, such as nitrogen and phosphorus, to host plants and produce growth-promoting substances, such as phytohormones, that enhance plant growth and development. In contrast, intercellular endophytic fungi can produce secondary metabolites that protect host plants from pathogens or herbivores (Deshmukh et al., 2006). In the present study, we observed both intercellular and intracellular colonization by fungal hyphae in the root tissues of hairy vetch and soybean plants.

Many different types of endophytic fungi can colonize intercellular or intracellular spaces in the root tissues of plants. However, we have been unable to identify any specific patterns related to the host specificity of the fungal species and colonization rate. Some fungal structures, such as vesicles, were observed only upon inoculation with specific isolates. The presence of similar fungal structures in soybean root tissues inoculated with the same endophytic fungi suggests the compatibility of the endophytic fungi isolated from hairy vetch plants with those from soybean plants (**Figure 6**).

## 5 Conclusion

This is the first study of the diversity and functional characteristics of culturable endophytic fungi associated with hairy vetch plants in Japan. The results indicated that the isolated fungi belong to three phyla, with Ascomycota and Basidiomycota predominating. The majority of isolates showed the potential to produce IAA and siderophores, as well as phosphate- and potassium-solubilizing activities. Certain isolates exhibited significant potential to promote the growth of both hairy vetch and soybean, which have not been previously reported as PGPFs. Inoculation of hairy vetch and soybean plants with these fungi significantly increased shoot and root growth in a host-specific manner. These findings highlight the importance of symbiotic associations between fungal endophytes and plants and illustrate the possibility of leveraging their beneficial attributes to enhance crop productivity and sustainability. Further exploration of the underlying mechanisms of these interactions holds promise for the development of innovative microbial-based strategies aimed at optimizing agricultural practices.

## Data Availability

The datasets presented in this study can be found in online repositories. The names of the repository/repositories and accession number(s) can be found in the article/**Supplementary Material**.
