# Supplementary material for: Diversity and plant growth promotion potential of endophytic fungi isolated from hairy vetch in Japan
Source: Front Plant Sci. 2024 Dec 19;15:1476200. doi: 10.3389/fpls.2024.1476200 (PMC11693453; doi:10.3389/fpls.2024.1476200)
Supplement: Supplementary file 1 [file DataSheet1.pdf]

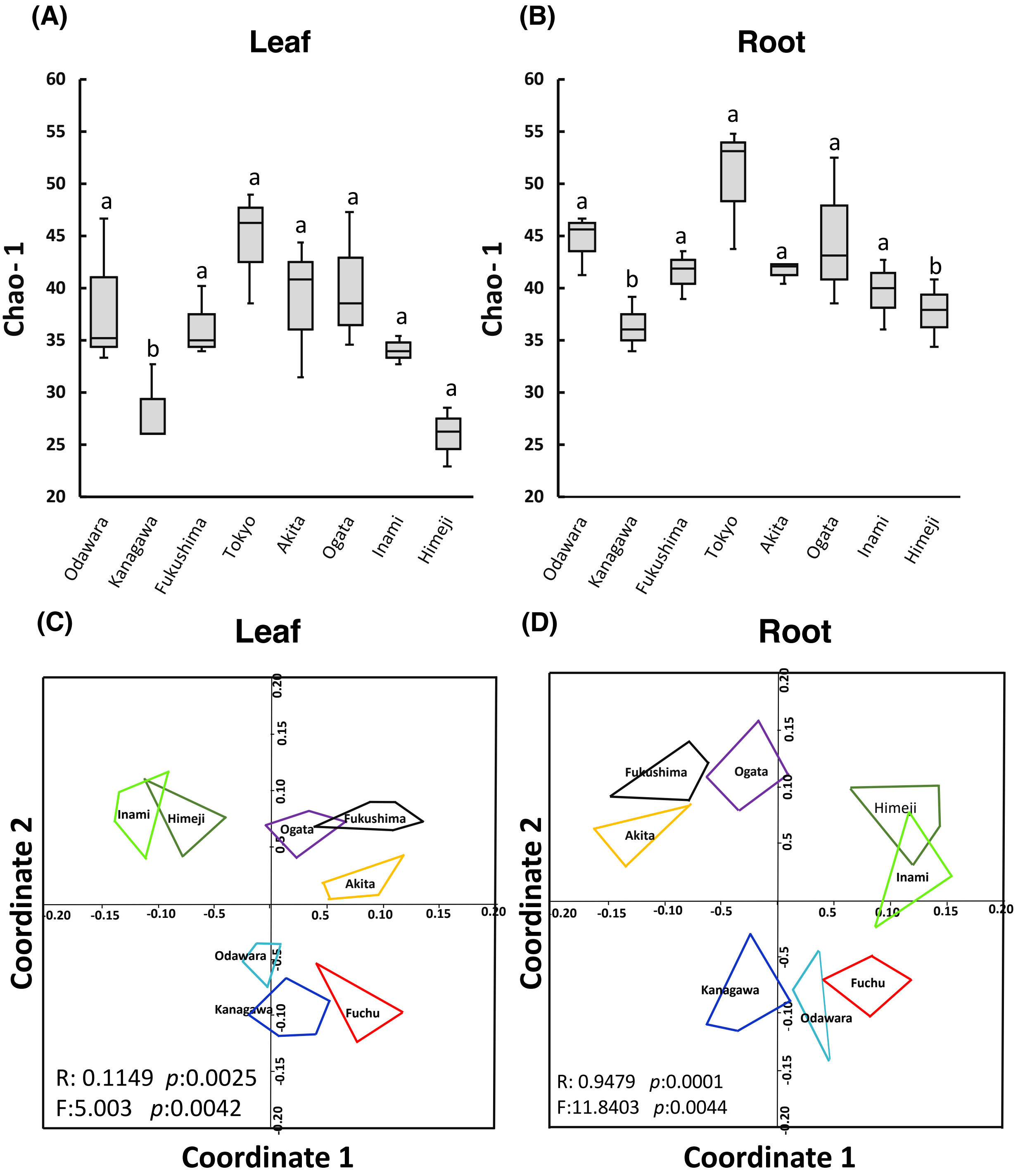

Supplementary Figure S1. Fungal endophyte biodiversity analysis. The effect of different host tissues (leaf and root) and sampling locations on fungal endophyte biodiversity as measured by **(A)** Chao1 estimator. Within each figure, different letters (a, b) above the bars indicate significant differences ($p < 0.05$, ANOVA and Tukey's HSD tests). **(B)** Nonmetric multidimensional scaling (NMDS) plots for cluster analyses of the fungal endophyte community were generated with Jaccard's index. The ANOSIM statistic R and the PERMANOVA statistic F values and the corresponding p values indicating the significance of dissimilarity were obtained by permutation of group membership, with 9999 replicates.

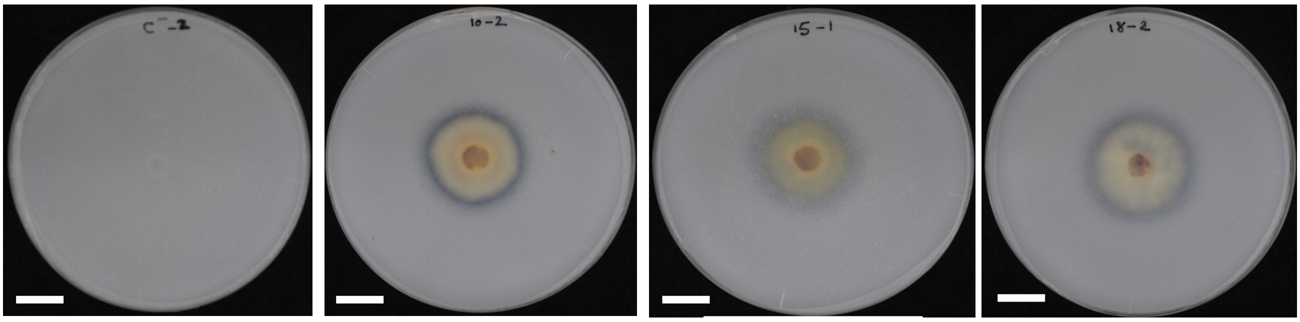

Control **FKR10** **AKR15** **FCL18**

Supplementary Figure S2. Phosphate solubilization activity of fungal isolates associated with *Vicia villosa* observed through the clearance zone formed on growth medium.

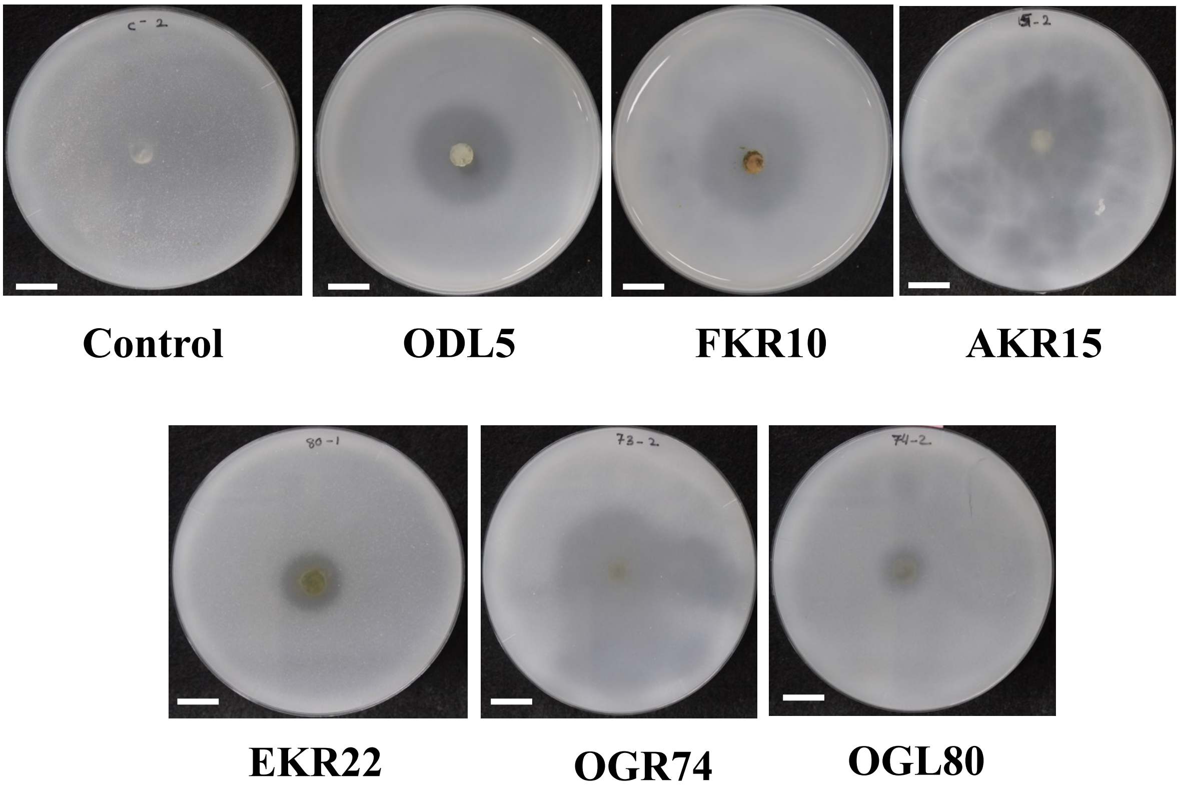

Supplementary Figure S3. Potassium solubilization activity of endophytic fungi associated with *Vicia villosa* observed through the clearance zone formed on growth medium (Aleksandrov + 0.2% sericite mica).

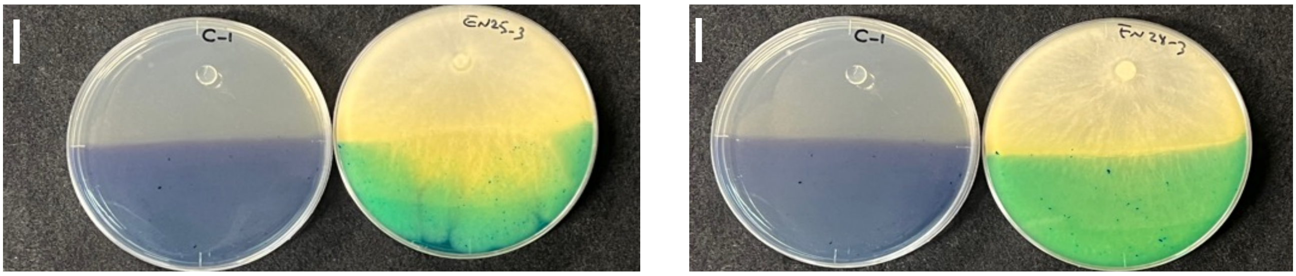

Control **AKL25** **Control** **FCR28**

Supplementary Figure S4. Plates contained PDA medium (yellow half) and CAS medium containing iron (blue half). Growth of the endophytic fungi on CAS agar plates with and without additional iron.

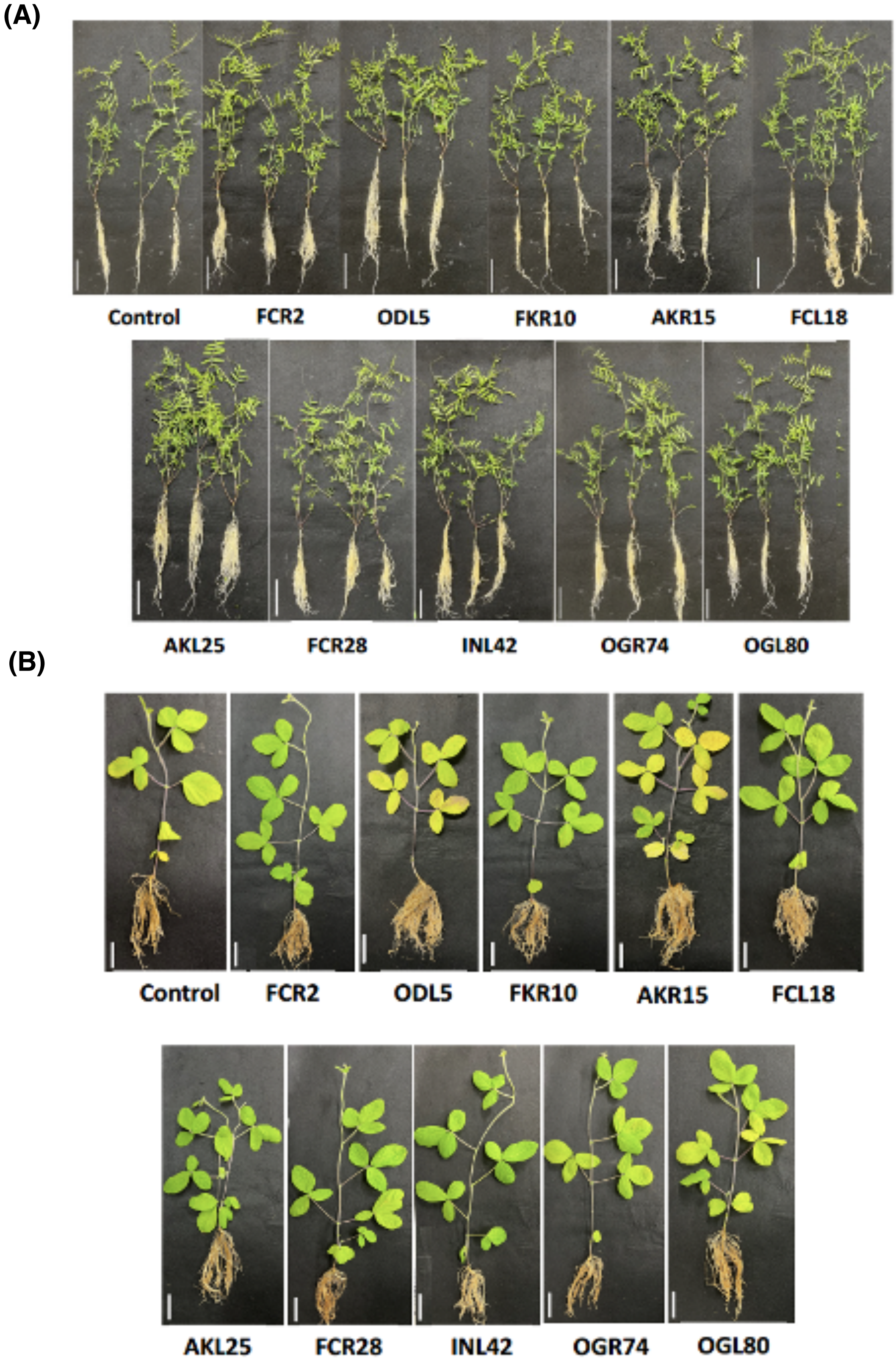

Supplementary Figure S5. The effect of endophytic fungi associated with hairy vetch (*Vicia villosa*) on (A) hairy vetch and (B) soybean plant growth 20 days after inoculation, scale bar =5 cm.
