# Supplementary material for: Diversity and plant growth promotion potential of endophytic fungi isolated from hairy vetch in Japan
Source: Front Plant Sci. 2024 Dec 19;15:1476200. doi: 10.3389/fpls.2024.1476200 (PMC11693453; doi:10.3389/fpls.2024.1476200)
Supplement: Supplementary file 2 [file Table1.pdf]

Supplementary Table 1. The genetic distribution of isolated endophytes from hairy vetch.

No	Isolate code	Scientific name	Accession number	Region	Tissue
1	AKR1	*Alternaria* sp.	OR004603	Akita	Root
2	FCR2	*Penicillium simplicissimum*	OR004604	Fuchu	Root
3	HML3	*Aspergillus fumigatus*	OR004605	Himeji	Leaf
4	INR4	*Trichoderma* sp.	OR004606	Inami	Root
5	ODL5	*Stemphylium lycopersici*	OR004607	Odawara	Leaf
6	OGR6	*Clonostachys* sp.	OR004608	Odawara	Root
7	KNL7	*Talaromyces pinophilus*	OR004609	Kanagawa	Leaf
8	FKR8	*Alternaria solani*	OR004610	Fukushima	Root
9	FCR9	*Botryosphaeria dothidea*	OR004611	Fuchu	Root
10	FKR10	*Trichoderma koningiopsis*	OR004612	Kanagawa	Root
11	AKL11	*Penicillium sp.*	OR004613	Akita	Leaf
12	HMR12	*Aspergillus* sp.	OR004614	Himeji	Root
13	FCL13	*Penicillium* sp.	OR004615	Fuchu	Leaf
14	INR14	*Trichoderma* sp.	OR004616	Inami	Root
15	AKR15	*Trichoderma koningii*	OR004617	Akita	Root
16	ODL16	*Curvularia trifolii*	OR004618	Odawara	Leaf
17	OGL17	*Trichoderma* sp.	OR004619	Ogata	Leaf
18	FCL18	*Trichoderma hamatum*	OR004620	Fuchu	Leaf
19	FKL19	*Clonostachys* sp.	OR004621	Fukushima	Leaf
20	FCR20	*Phoma herbarum*	OR004622	Fuchu	Root
21	INL21	*Aspergillus brunneoviolaceus*	OR004623	Inami	Leaf
22	KNR22	*Alternaria tamaricis*	OR004624	Kanagawa	Root
23	OGR23	*Cladosporium anthropophilum*	OR004625	Ogata	Root
24	FKL24	*Didymella glomerata*	OR004626	Fukushima	Leaf
25	AKL25	*Penicillium griseofulvum*	OR004627	Akita	Leaf
26	HML26	*Penicillium* sp.	OR004628	Himeji	Leaf
27	KNL27	*Fusarium verticillioides*	OR004629	Kanagawa	Leaf
28	FCR28	*Trichoderma atrobrunneum*	OR004630	Fuchu	Root
29	FKR29	*Alternaria seleniiphila*	OR004631	Fukushima	Root
30	OGR30	*Penicillium* sp.	OR004632	Ogata	Root
31	INL31	*Penicillium* sp.	OR004633	Inami	Leaf
32	FKL32	*Clonostachys rosea*	OR004634	Fukushima	Leaf
33	FCR33	*Talaromyces sp*	OR004635	Fuchu	Root
34	ODL34	*Epicoccum nigrum*	OR004636	Odawara	Leaf
35	HML35	*Trichoderma* sp.	OR004637	Himeji	Leaf
36	AKR36	*Trichoderma hamatum*	OR004638	Akita	Root
37	OGL37	*Trichoderma* sp.	OR004639	Ogata	Leaf
38	FKR38	*Talaromyces* sp.	OR004640	Fukushima	Root
39	KNL39	*Diaporthe* sp.	OR004641	Kanagawa	Leaf
40	FCL40	*Mucor hiemalis*	OR004642	Fuchu	Leaf
41	ODR41	*Fusarium* sp.	OR004643	Odawara	Root
42	INL42	*Phoma* sp.	OR004644	Inami	Leaf
43	FKL43	*Clonostachys rosea*	OR004645	Fukushima	Leaf
44	FCR44	*Mucor* sp.	OR004646	Fuchu	Root
45	AKL45	*Fusarium graminearum*	OR004647	Akita	Leaf
46	INL46	*Pestalotiopsis sp.*	OR004648	Inami	Leaf
47	KNR47	*Fusarium verticillioides*	OR004649	Kanagawa	Root

48	FCR48	*Fusarium avenaceum*	OR004650	Fuchu	Root
49	FKL49	*Curvularia lunata*	OR004651	Fukushima	Leaf
50	ODR50	*Fusarium tricinctum*	OR004652	Odawara	Root
51	KNL51	*Phialocephala bamuru*	OR004653	Kanagawa	Leaf
52	OGR52	*Penicillium* sp.	OR004654	Ogata	Root
53	FKL53	*Talaromyces pinophilus*	OR004655	Fukushima	Leaf
54	FCR54	*Penicillium ochrochloron*	OR004656	Fuchu	Root
55	AKL55	*Hongkongmyces snookiorum*	OR004657	Akita	Leaf
56	FKR56	*Chaetomium globosum*	OR004658	Fukushima	Root
57	KNL57	*Cladosporium uredinicola*	OR004659	Kanagawa	Leaf
58	INR58	*Fusarium* sp.	OR004660	Inami	Root
59	AKL59	*Termitomyces aurantiacus*	OR004661	Akita	Leaf
60	FKL60	*Phoma* sp.	OR004662	Fukushima	Leaf
61	ODR61	*Talaromyces assiutensis*	OR004663	Odawara	Root
62	AKR62	*Schizophyllum commune*	OR004664	Akita	Root
63	HML63	*Aspergillus oryzae*	OR004665	Himeji	Leaf
64	KNR64	*Rhizopus arrhizus*	OR004666	Kanagawa	Root
65	ODL65	*Alternaria* sp.	OR004667	Odawara	Leaf
66	FKR66	*Cladosporium halotolerans*	OR004668	Fukushima	Root
67	FCL67	*Diaporthe phragmitis*	OR004669	Fuchu	Leaf
68	INR68	*Alternaria ricini*	OR004670	Inami	Root
69	AKL69	*Ceratobasidium* sp.	OR004671	Akita	Leaf
70	OGR70	*Chaetomium globosum*	OR004672	Ogata	Root
71	FCL71	*Pleosporales* sp.	OR004673	Fuchu	Leaf
72	KNL72	*Penicillium oxalicum*	OR004674	Kanagawa	Leaf
74	OGR74	*Aspergillus nomiae*	OR004676	Ogata	Root
75	FKL75	*Fusarium proliferatum*	OR004677	Fukushima	Leaf
76	AKL76	*Diaporthe nobilis*	OR004678	Akita	Leaf
77	INL77	*Talaromyces muroii*	OR004679	Inami	Leaf
78	FCL78	*Diaporthe amygdali*	OR004680	Fuchu	Leaf
79	KNR79	*Diaporhte* sp.	OR004681	Kanagawa	Root
80	OGL80	*Alternaria alternata*	OR004682	Ogata	Leaf

Supplementary Table 2: Pearson's correlation analysis (PCA) between properties and the fungal communities.

	pH	STP	SMO	TN	TK	EC	NH4-N	*Penicillium*	*Alternaria*	*Trichoderma*	*Fusarium*	*Cladosporium*	*Talaromycetes*	*Clonostachys*	*Aspergillus*
pH		0.13309	0.2611	0.48304	0.55207	0.1292	0.32061	0.01942	0.60348	0.43388	0.82127	0.37943	0.67158	0.28642	0.6371
STP	0.57842		0.99062	0.69504	0.33861	0.096094	0.56206	0.30174	0.93323	0.5892	0.88822	0.53266	0.62198	0.17167	0.80481
SMO	0.4518	0.005004		0.10582	0.86237	0.31581	0.83698	0.93205	0.80494	0.8699	0.17288	0.0054465	0.80958	0.46719	0.33364
TN	0.29186	-0.16564	0.61338		0.096678	0.83624	0.71922	0.3338	0.9522	0.73305	0.56127	0.0095975	0.93728	0.76257	0.6077
TK	0.249	-0.39067	0.073666	0.62623		0.73201	0.31042	0.14874	0.23001	0.029546	0.084404	0.19127	0.46273	0.25958	0.16123
EC	-0.58314	-0.62708	0.40788	-0.08779	0.14495		0.038522	0.42577	0.27442	0.78321	0.38015	0.7164	0.97448	0.027034	0.5359
NH4-N	0.40421	0.24296	0.087389	0.15208	0.41204	-0.73311		0.2854	0.0033562	0.098566	0.85638	0.7248	0.7911	0.41908	0.095805
Penicillium	0.79091	0.4188	0.036273	0.39426	0.56014	-0.32928	0.43179		0.53649	0.17135	0.099096	0.6449	0.50497	0.83926	0.07919
Alternaria	0.21832	-0.035639	-0.1048	-0.025506	0.47881	-0.44072	0.886444	0.25849		0.080333	0.6788	0.98815	0.96917	0.59773	0.12855
Trichoderma	0.32386	-0.22675	-0.069609	0.14437	0.75731	-0.11668	0.62353	0.53553	0.6511		0.36574	0.54513	0.063659	0.43644	0.19748
Fusarium	0.095911	-0.059756	-0.53392	0.24343	0.64465	0.36064	0.076897	0.62277	0.17484	0.37088		0.68487	0.94137	0.34191	0.042155
Cladosporium	-0.36114	0.26.84	-0.86583	-0.8367	-0.51525	0.15365	-0.15138	-0.19421	-0.0063185	-0.25321	0.17139		0.48863	0.78291	0.69399
Talaromycetes	0.17894	-0.20749	0.10227	0.033477	0.30492	0.013613	0.11235	0.27801	-0.016447	0.67974	-0.031292	-0.28831		0.28363	0.99808
Clonostachys	-0.43096	-0.53519	-0.30203	0.12802	0.45308	0.76488	-0.33379	-0.086153	-0.2217	0.32216	0.38823	-0.11684	0.43322		0.83938
Aspergillus	0.19872	0.10487	-0.39438	0.21584	0.54631	0.25885	0.6275	0.65295	0.58394	0.50917	0.7243	0.16624	0.0010262	0.086089	

Supplementary Table 3. PGP traits of culture- dependent isolates associated with hairy vetch.

Isolates	Species	IAA (mg L−1)	P solubilization (mm)	K solubilization (mm)	Siderophore production
AKR1	*Alternaria sp.*		2.20± 0.03	0	0
FCR2	*Penicillium simplicissimum*	15.1± 0.13	0	0	37.0± 0.12
HML3	*Aspergillus fumigatus*	0	0	0	0
INR4	*Trichoderma sp.*	0	0	0	30.3± 0.10
ODL5	*Stemphylium lycopersici*	8.00± 0.11	0	2.85± 0.03	39.4± 0.07
OGR6	*Clonostachys sp.*		1.10± 0.05	0	0
KNL7	*Talaromyces pinophilus*	0	0	2.0± 0.07	0
FKR8	*Alternaria solani*	0	0	0	0
FCR9	*Botryosphaeria dothidea*	0	0	0	5.90± 0.06
FKR10	*Trichoderma koningiopsis*	9.55± 0.04	1.16± 0.05	4.24± 0.02	0
AKL11	*Penicillium sp.*	12.5± 0.02	0	0	0
HMR12	*Aspergillus sp.*	0	0	0	25.83± 0.02
FCL13	*Penicillium sp.*	0	0	0	0
INR14	*Trichoderma sp.*	10.2± 0.06	0	0	0
AKR15	*Trichoderma koningii*	6.23± 0.03	1.25± 0.02	4.26± 0.06	61.0± 0.04
ODL16	*Curvularia trifolii*	2.55± 0.01	0	0	0
OGL17	*Trichoderma sp.*	5.12± 0.03	0	0	0
FCL18	*Trichoderma hamatum*	6.11± 0.10	1.27± 0.02	0	60.3± 0.10
FKL19	*Clonostachys sp.*	0	0	0	0
FCR20	*Phoma herbarum*	2.15± 0.04	0	0	0
INL21	*Aspergillus brunneoviolaceus*	0	0	0	0
EKR22	*Alternaria tamaricis*	0	0	2.12± 0.03	0
OGR23	*Cladosporium anthropophilum*	15.1± 0.06	0	0	15.5± 0.02
FKL24	*Didymella glomerata*	0	0	0	28.8± 0.05
AKL25	*Penicillium griseofulvum*	10.2± 0.23	0	0	64.7± 0.07
HML26	*Penicillium sp.*	0	0	0	16.5± 0.03
KNL27	*Fusarium verticillioides*	0	0	0	25.4± 0.02
FCR28	*Trichoderma atrobrunneum*	1.35± 0.05	1.21± 0.06	7.92± 0.03	63.8± 0.03
FKR29	*Alternaria seleniiphila*	10.5± 0.03	0	1.68± 0.02	0
OGR30	*Penicillium sp.*	7.15± 0.04	0	2.99± 0.06	0
INL31	*Penicillium sp.*	8.00 0.11	0	0	40.2± 0.03
FKL32	*Clonostachys rosea*	3.15± 0.02	0	0	15.5± 0.08
FCR33	*Talaromyces sp*	1.45± 0.06	0	0	25. 8± 0.04
ODL34	*Epicoccum nigrum*	0	0	0	30. 2± 0.05
HML35	*Trichoderma sp.*	1.55± 0.03	0	0	0
AKR36	*Trichoderma hamatum*	5.35± 0.12	0	0	0
OGL37	*Trichoderma sp.*	0	0	0	0
FKR38	*Talaromyces sp.*	2.35± 0.06	0	0	0
KNL39	*Diaporthe sp.*	1.14± 0.12	0	0	32. 8± 0.03
FCL40	*Mucor hiemalis*	0	0	0	0
ODR41	*Fusarium sp.*	0	1.28± 0.02	0	0
INL42	*Phoma sp.*	5.80± 0.03	0	0	33.0± 0.02
FKL43	*Clonostachys rosea*	0	0	0	13.0± 0.02

FCR44	*Mucor sp.*	8.35± 0.06	0	0	20.0± 0.04
AKL45	*Fusarium graminearum*	0	0	0	0
INL46	*Pestalotiopsis sp.*	2.30± 0.04	0	0	61.1± 0.02
KNR47	*Fusarium verticillioides*	0	0	0	0
FCR48	*Fusarium avenaceum*	2.35± 0.06	0	0	0
FKL49	*Curvularia lunata*	4.20± 0.05	0	0	23. 4± 0.03
ODR50	*Fusarium tricinctum*	3.5± 0.03	0	0	0
KNL51	*Phialocephala bamuru*	0	0	0	0
OGR52	*Penicillium sp.*	2.00± 0.02	0	0	0
FKL53	*Talaromyces pinophilus*	1.50± 0.05	0	0	0
FCR54	*Penicillium ochrochloron*	0	0	0	61. 3± 0.06
AKL55	*Hongkongmyces snookiorum i*	0	0	0	0
FKR56	*Chaetomium globosum*	0	0	0	0
KNL57	*Cladosporium uredinicola*	5.20± 0.03	0	0	15. 5± 0.05
INR58	*Fusarium sp.*	3.55± 0.01	0	0	0
AKL59	*Termitomyces aurantiacus*	4.23± 0.05	0	0	28. 0± 0.03
FKL60	*Phoma sp.*	0	0	0	31. 0± 0.07
ODR61	*Talaromyces assiutensis*	0	0	0	0
AKR62	*Schizophyllum commune*	0	0	0	18. 0± 0.02
HML63	*Aspergillus oryzae*	0	0	0	22. 4± 0.06
KNR64	*Rhizopus arrhizus*	0	0	0	20. 0± 0.06
ODL65	*Alternaria sp.*	2.00± 0.01	1.23± 0.06	0	18. 6± 0.03
FKR66	*Cladosporium halotolerans*	0	0	0	0
FCL67	*Diaporthe phragmitis*	0	0	2.55± 0.05	0
INR68	*Alternaria ricini*	7.48± 0.05	0	0	0
AKL69	*Ceratobasidium sp.*	0	0	0	32.0± 0.01
OGR70	*Chaetomium globosum*	3.00± 0.04	0	0	27.0± 0.07
FCL71	*Pleosporales sp.*	0	0	0	27.5± 0.04
KNL72	*Penicillium oxalicum*	0	0	0	30.0± 0.04
OGR74	*Pseudopithomyces palmicola*	5.06± 0.04	0	1.51± 0.05	0
FKL75	*Aspergillus nomiae*	3.25± 0.05	0	0	56.7± 0.02
AKL76	*Fusarium proliferatum*	2.50± 0.06	0	0	0
INL77	*Diaporthe nobilis*	0	0	3.33± 0.06	35.7± 0.06
FCL78	*Talaromyces muroii*	5. 35± 0.03	1.20± 0.04	0	18.7 ± 0.06
KNR79	*Diaporthe amygdali*	0	1.18± 0.07	0	0
OGL80	*Diaporhte sp.*	2.70± 0.07	0	2.11± 0.01	0
